# ACE-Inhibitory Peptides from Yanbian Cattle Hemoglobin: Screening, Kinetics, and Molecular Dynamics Simulation

**DOI:** 10.3390/foods15081414

**Published:** 2026-04-17

**Authors:** Shihan Yang, Tingting Gao, Bowen Qin, Chenguang Li, Chunxiang Piao, Mingxun Cui, Hongmei Li, Baide Mu, Juan Wang, Tingyu Li, Qingwei Jiang, Aihui Lv, Guanhao Li

**Affiliations:** 1 College of Agriculture, Yanbian University, Yanji 133000, China; yangshihan2026@163.com (S.Y.); 13704329945@163.com (T.G.); qinbw0101@163.com (B.Q.); master0216@163.com (C.L.); cxpiao@ybu.edu.cn (C.P.); 0000001384@ybu.edu.cn (M.C.); 13044337020@163.com (H.L.); mubaide@ybu.edu.cn (B.M.); wangjuan@ybu.edu.cn (J.W.); 0000008801@ybu.edu.cn (T.L.); 2 Key Innovation Laboratory for Deep and Intensive Processing of Yanbian High Quality Beef, Ministry of Agriculture and Rural Affairs, Yanbian University, Yanji 133000, China; youaihui@hotmail.com; 3 Key Laboratory of Natural Medicines of the Changbai Mountain, Ministry of Education, College of Pharmacy, Yanbian University, Yanji 133000, China; jiangqingwei@thpharms.com; 4 Yanbian Animal Husbandry Development Group, Yanji 133000, China

**Keywords:** Yanbian yellow cattle, bovine blood, ACE inhibitory peptides, molecular docking, molecular dynamics simulation

## Abstract

The global burden of hypertension continues to rise, highlighting an urgent need for effective therapeutic strategies. Angiotensin-converting enzyme (ACE) is central to blood pressure regulation, but commonly used synthetic ACE inhibitors often have adverse side effects, spurring the search for safer natural alternatives. The aim of this study was to investigate Yanbian cattle hemoglobin as a novel precursor for ACE inhibitory peptides. The <1 kDa fraction was identified as exhibiting the highest inhibitory activity through the systematic screening of hydrolysates across multiple molecular weight ranges. LC-MS/MS analysis identified 1980 peptides, of which four were selected for further experiments. Solid-phase synthesis confirmed that NFGYDL exhibited the strongest ACE inhibition (IC_50_ = 54.95 μM). Inhibition kinetics showed FHDYL acted as a mixed-type inhibitor, DLGHF and NFGYDL as competitive inhibitors and GFHLD as a non-competitive inhibitor. Molecular dynamics simulations validated the stable binding of these bovine blood-derived peptides to the ACE complex. HUVEC functional assays demonstrated that four peptides significantly increased angiotensin II-induced nitric oxide production and endothelin-1 levels, suggesting their potential antihypertensive activity. These findings suggested that bovine blood is a promising natural source of ACE-inhibitory peptides and holds potential for application as a functional component in functional foods targeting hypertension management.

## 1. Introduction

Hypertension is a pervasive global health challenge, affecting over one billion individuals worldwide. It serves as a primary risk factor for severe cardiovascular and cerebrovascular events, such as stroke and coronary heart disease [1]. Angiotensin-converting enzyme (ACE) plays a pivotal role in blood pressure regulation by catalyzing the conversion of angiotensin I to angiotensin II (Ang II). As a potent vasoconstrictor, Ang II stimulates the release of aldosterone and antidiuretic hormone, and promotes sodium reabsorption, ultimately leading to elevated blood pressure. Consequently, inhibiting ACE activity is considered a primary therapeutic strategy for the prevention and management of hypertension [2]. Currently, two types of drugs used to treat hypertension are synthetic Angiotensin-Converting Enzyme (ACE) inhibitors and food-derived bioactive peptides. Although unnatural small molecule ACE inhibitors, such as captopril, enalapril and benazepril. While highly effective, their long-term administration is frequently associated with adverse side effects, including headache, insomnia, fatigue, and hyperkalemia [3]. Compared with synthetic inhibitors, naturally obtained inhibitors usually exhibit better stability and fewer side effects, and they could be potential drugs for treating hypertension for a long time [4]. Therefore, it is of great significance to find natural inhibitors as safer alternative drugs for the treatment of hypertension [5].

As one of the five premier indigenous cattle breeds in China, Yanbian cattle are renowned for their tender, juicy, and highly nutritious meat. A significant byproduct of Yanbian cattle slaughtering and processing is blood, which constitutes 5.0% to 6.0% of the live weight. It is a rich protein source, containing 17% to 20% protein and exhibiting a balanced amino acid profile. Hemoglobin is the dominant protein in erythrocytes, accounting for over 90% of the erythrocyte protein and approximately 80% of the total blood protein. This high abundance establishes bovine blood as a high-quality and sustainable protein resource for functional food applications. Hemoglobin features a compact quaternary structure that conceals numerous hydrophobic amino acid sequences. These sequences can be released through enzymatic hydrolysis to generate structurally diverse peptides. Additionally, hemoglobin is rich in hydrophobic and branched-chain amino acids. This specific amino acid composition makes it an ideal precursor for ACE inhibitory peptides, thereby making it worthy of dedicated investigation [6].

However, the comprehensive utilization of bovine blood remains restricted by several inherent challenges. Its distinctive odor reduces sensory palatability, whereas its in vivo digestibility and bioavailability are relatively low. Moreover, practical difficulties in large scale collection and preservation, together with potential environmental pollution risks, further limit its industrial application. Recent studies have confirmed its considerable potential as a source of bioactive peptides, such as antioxidative [7], andantimicrobial peptides [8]. Oral administration represents the most feasible and promising route for bioactive peptides derived from food. Nevertheless, the gastrointestinal digestive environment strongly affects their in vivo stability and activity. Orally delivered ACE inhibitory peptides are exposed to acidic gastric fluid and multiple digestive enzymes, including pepsin, trypsin, and chymotrypsin, which may cause peptide degradation, structural damage, and consequent activity loss [4]. Therefore, screening structurally stable and highly potent ACE inhibitory peptides from bovine hemoglobin that are resistant to gastrointestinal digestion is of great practical significance for developing orally available natural antihypertensive foods or functional components. Collectively, these studies indicate that bovine blood is a promising natural source of ACE inhibitory peptides, offering a sustainable strategy for its high value utilization in functional foods and nutraceuticals.

Computational virtual screening technology can rapidly predict the interactions between small molecules and target proteins, providing an efficient and reliable theoretical basis for the preliminary evaluation of the potential efficacy of bioactive compounds [9]. Molecular docking is the most commonly used method to study the interaction between ligands and receptor proteins, which can be applied to predict the binding status and interaction forces between them [10]. Molecular Dynamics can simulate dynamically evolving visual images, thereby obtaining information such as the secondary structure and hydrogen bonds of proteins and complexes formed by proteins and ligands [11]. Consequently, molecular dynamics simulations serve as a critical validation tool to assess the structural stability and binding persistence of complexes initially identified through molecular docking. Growing recognition of this synergistic approach has led researchers to integrate docking with molecular dynamics simulations, offering a comprehensive theoretical framework to elucidate mechanistic details at atomic resolution. Qi et al. conducted molecular dynamics simulations on the complex of ACE and ligands after molecular docking. By calculating the root mean square deviation, root mean square fluctuation, and radius of gyration, they demonstrated that the complex between ACE and LAAHFAR after molecular docking was stable [12]. Human umbilical vein endothelial cells (HUVECs), which play a pivotal role in cardiovascular homeostasis by secreting key vasoactive mediators such as nitric oxide (NO) and endothelin 1 (ET-1), represent an ideal in vitro model for investigating the antihypertensive mechanisms of bioactive peptides. For instance, Huang et al. demonstrated that two peptides derived from yeast, GPAGPAGPR and GPPGPPGL, significantly enhanced nitric oxide production at a concentration of 600 μg/mL, suggesting their potential as antihypertensive agents [13]. Furthermore, Wei et al. discovered several ACE inhibitory peptides, including VE, FEF, and WEEF, from fermented soybean curd. All of them could significantly suppress endothelin 1 secretion in human umbilical vein endothelial cells, and WEEF showed the strongest inhibitory activity at 400 μmol/L [14].

Studies on ACE-inhibitory peptides derived from Yanbian cattle hemoglobin remain scarce. Most existing reports focus on the preparation and activity evaluation of bovine blood peptides, while their endothelial protective effects and underlying antihypertensive mechanisms have not been systematically elucidated. This study aims to prepare and identify novel ACE inhibitory peptides from Yanbian cattle hemoglobin, a characteristic local livestock resource that has been rarely investigated. By combining enzymatic preparation, molecular docking, molecular dynamics simulation, and cellular functional evaluation, this work systematically characterizes the ACE inhibitory activity, binding interaction, and vascular endothelial regulatory effects of the obtained peptides. This study not only identifies novel potential ACE inhibitory peptides, but also provides a theoretical basis and scientific support for the high-value utilization of Yanbian cattle blood resources.

## 2. Materials and Methods

### 2.1. Materials

Fresh Yanbian yellow cattle blood was procured from Baoren Slaughterhouse (Yanji, China). Alkaline protease, Activated carbon (particle size 0.075 mm, 200 mesh, CAS No. 7440-44-0), Sephadex G-15, and CCK-8 kit were obtained from Solarbio Biotechnology (Beijing, China). Rabbit lung angiotensin-converting enzyme (ACE, 0.1 U/mL) and N-[3-(2-Furyl)acryloyl]-Phe-Gly-Gly (FAPGG) was obtained from Yuanye Biotechnology (Shanghai, China). All other chemicals and reagents, including hydrochloric acid, were acquired from commercial suppliers, sodium hydroxide, formaldehyde, and acetonitrile, were of analytical reagent grade. HUVECs were acquired from Yimo Biotechnology (Xiamen, China). The NO detection kit and ET-1 ELISA kit were purchased from Nanjing Jiancheng Bioengineering Institute (Nanjing, China) and Shanghai Jining Biotechnology Co. (Shanghai, China).

### 2.2. Extraction of the Crude Peptides

Fresh Yanbian cattle blood was collected and immediately anticoagulated with 0.5% (*w*/*v*) sodium citrate. The blood cells were subjected to repeated washing and freeze-thaw cycles, followed by ultrasonication at a 1:4 (*w*/*v*) cell-to-water ratio for cellular disruption. The resulting lysate was centrifuged to isolate the hemoglobin fraction, which was subsequently lyophilized and stored. The obtained powder was dissolved in deionized water at a solid-to-liquid ratio of 50 g/L, and protease 4000 U/g was added. Sodium hydroxide solution was then added dropwise to adjust the pH to 9, and the enzymatic hydrolysis was carried out at 45 °C for 5 h. The enzymatic reaction was terminated by heat inactivation, and the hydrolysate was centrifuged to remove insoluble residues. The clarified supernatant was decolorized with activated carbon and lyophilized to obtain the bovine hemoglobin-derived ACE inhibitory peptides. The final peptide powder was stored at −20 °C.

### 2.3. Ultrafiltration

The bovine hemoglobin-derived ACE inhibitory peptides were sequentially fractionated by ultrafiltration using 3 kDa and 1 kDa molecular weight cut-off (MWCO) membranes at 4 °C, with minor modifications according to a previous method [15]. Three fractions were collected and designated as U1 (>3 kDa), U2 (1–3 kDa), and U3 (<1 kDa). All fractions were lyophilized, and their ACE inhibitory activities and peptide contents were quantitatively determined. The fraction with the highest ACE inhibitory activity was selected for further purification.

### 2.4. Gel Filtration Chromatography

The lyophilized U3 fraction (<1 kDa) was dissolved in deionized water and filtered through a 0.45 μm membrane to remove insoluble impurities. The sample was loaded onto a Sephadex G-15 column pre-equilibrated with ultrapure water and eluted at a flow rate of 1 mL/min [16]. Elution was monitored at 220 nm for peptide bond absorption. Distinct peak fractions were collected separately, lyophilized, and analyzed for ACE inhibitory activity and peptide content to identify the most bioactive fraction.

### 2.5. Determination of ACE-Inhibitory Activity

The ACE inhibitory activity was determined in 96-well plates according to the method described by Qiu et al. [17] with minor modifications. Briefly, 80 mmol/L HEPES buffer containing 0.3 mol/L NaCl (pH 8.3) was used as the reaction buffer. The substrate N-[3-(2-furyl)acryloyl]-Phe-Gly-Gly (FAPGG) was prepared in the same HEPES buffer. To ensure the accuracy and reliability of the assay, a substrate blank control without ACE. Each sample was tested in at least three independent replicates. Each reaction mixture in the sample well consisted of 40 μL peptide solution, 50 μL FAPGG and 10 μL ACE solution, while the blank control contained 40 μL HEPES buffer instead of peptide solution. Initial absorbance at 340 nm was recorded immediately using a microplate reader, followed by a second measurement after 30 min incubation at 37 °C. The inhibition rate was calculated using Equation.

$$ACE\;inhibition\;rate\;(\%)=\frac{{\Delta A}_{control}-{\Delta A}_{sample}}{{\Delta A}_{control}}\times 100$$

where ΔAcontrol and ΔAsample are defined as the absorbance decrease over 30 min in the blank and sample groups, respectively.

Using the concentration of the ACE inhibitory peptide as the abscissa (X-axis) and the corresponding ACE inhibition rate as the ordinate (Y-axis), a curve was plotted using Origin 2018b software. The IC_50_ value, defined as the peptide concentration required to achieve 50% ACE inhibition, was calculated from the regression equation obtained by curve fitting. Captopril was used as the positive control.

### 2.6. Determination of Peptide Content

The peptide content was determined using the o-phthaldialdehyde (OPA) method, a reagent commonly used for the quantitative detection of primary amines in peptides and proteins. The peptide content was determined according to the method described by Xing et al. [18] with minor modifications. The OPA working solution was prepared by dissolving 40 mg o-phthaldialdehyde in 1 mL methanol, then mixing with 25 mL borax buffer (100 mmol/L), 2.5 mL SDS (20%), and 100 μL β-mercaptoethanol, with final volume adjusted to 50 mL using deionized water. For analysis, 100 μL sample was reacted with 2 mL OPA reagent at room temperature for 2 min, followed by absorbance measurement at 340 nm. The peptide content was calculated using a standard curve (Figure S1).

The peptide content (%) was calculated as follows:

$$Peptidecontent\;(\%)=\frac{x\times V}{m\times 1000}\times 100$$

where, x = peptide concentration of sample solution (mg/mL), V = total volume of sample solution (100 mL), m = mass of sample (1 mg).

### 2.7. Screening and Analysis of Potential ACE Inhibitory Peptides

For LC-MS/MS analysis, protein samples were dissolved in 50 mM ammonium bicarbonate. The samples were reduced with 10 mM dithiothreitol (DTT) at 56 °C for 1 h, followed by alkylation with 20 mM iodoacetamide (IAM) in the dark at room temperature for 40 min. Excess IAM was quenched by adding an additional 10 mM DTT. Subsequently, the samples were digested with trypsin (enzyme-to-substrate ratio of 1:50, *w*/*w*) at 37 °C overnight. The resulting peptides were desalted using a C18 StageTip and vacuum-dried at 45 °C prior to MS analysis.

The dried sample was subsequently reconstituted in 40 μL of 0.1% (*v*/*v*) aqueous trifluoroacetic acid prior to LC-MS/MS analysis. The chromatographic separation was carried out using a system consisting of a pre-column coupled to an analytical column. The mobile phase comprised (A) 0.1% (*v*/*v*) formic acid in water and (B) 0.1% (*v*/*v*) formic acid in 80% (*v*/*v*) acetonitrile, delivered at a flow rate of 600 nL/min over a 66 min total runtime.

Sample analysis was performed on an integrated liquid chromatography-tandem mass spectrometry system consisting of an Easy-nLC 1200 HPLC system coupled to a Q Exactive quadrupole-Orbitrap mass spectrometer (Thermo Fisher Scientific, Waltham, MA, USA). Mass spectrometric detection was operated in positive electrospray ionization (ESI) mode, and peptide fragmentation was performed using higher-energy collisional dissociation (HCD). For comprehensive peptide profiling, MS data were acquired in DDA (data-dependent acquisition) mode: full MS scans were acquired at a resolution of 70,000 (*m*/*z* 100–1500), followed by HCD-MS/MS (28% normalized collision energy) of the top 20 most abundant ions at a resolution of 17,500. Ions with charge states ≥1 were selected for MS/MS fragmentation, and singly charged ions were not excluded. Raw data were processed and searched using MaxQuant (v1.5.5.1) against the Bos taurus proteome database (uniprotkb_proteome_Bos_taurus(9913)_2024_05_20) with the following parameters: fixed modification of carbamidomethyl (C); variable modifications of oxidation (M) and acetyl (Peptide N-term); enzyme specificity set as non-specific; peptide mass tolerance of 20 ppm; and fragment mass tolerance of 0.02 Da. All parameters were set in accordance with standard instrument protocols and proteomic experimental specifications to ensure the accuracy and reliability of the identification results. The LC–MS/MS proteomics data generated in this study have been publicly deposited in the ProteomeXchange Consortium via the iProX repository [19], with the official dataset accession number PXD076229.

### 2.8. Virtual Screening

The identified peptides were screened against the BIOPEP database to eliminate known ACE inhibitory sequences and focus on novel candidates. The remaining peptides were further evaluated for their bioactive potential via Peptide Ranker. Solubility was predicted using the Innovagen Peptide Property Calculator, toxicity was assessed with ToxinPred, and human intestinal absorption (HIA) was evaluated using admetSAR [20]. The final selection yielded novel peptides with high predicted bioactivity, excellent solubility, non-toxicity, efficient intestinal absorption, and strong blood-brain barrier permeability.

### 2.9. Synthesis of Peptides

The candidate peptides were commercially synthesized by Hongtai Biotechnology Co., Ltd. (Shanghai, China) using Fmoc solid-phase synthesis and were supplied as lyophilized powders. Prior to experimental use, molecular weight and purity were verified by MALDI-TOF mass spectrometry. Peptides meeting the ≥95% purity threshold (confirmed by chromatographic analysis) were advanced to further studies.

### 2.10. Molecular Docking

Molecular docking was performed with AutoDock Vina 1.2.3 [21] following standardized preparation protocols for protein and peptide structures. The crystal structure of human ACE was obtained from the Protein Data Bank (PDB ID: 1O86). The ACE structure was preprocessed using PyMOL 2.5.5 by removing crystallographic water molecules, ions, and non-relevant ligands, while the zinc ion at the catalytic active site was retained. Polar hydrogen atoms were added, and Gasteiger charges were assigned to the protein structure. The docking grid box was centered at the ACE active site with center_x = −13.36, center_y = −8.05, center_z = 21.53, and the grid dimensions were set as size_x = 20, size_y = 20, size_z = 20. The default scoring function embedded in AutoDock Vina 1.2.3 was applied to evaluate the binding affinity between ACE and the peptides. Both the receptor and ligand structures were converted to PDBQT format via ADFRsuite 1.0 [22] to ensure software compatibility. Docking calculations were performed with an exhaustiveness parameter of 16 for sufficient conformational sampling, with other parameters set as default. The dominant binding conformation was selected according to the highest binding affinity score (binding free energy, kcal/mol). Molecular interactions were further analyzed and visualized using PyMOL 2.5.5.

### 2.11. Molecular Dynamics Simulations

All-atom molecular dynamics simulations of the docked peptide-protein complexes were conducted using AMBER 24. The ff14SB force field was employed for both the protein and peptide. The system was prepared by adding hydrogen atoms via the LEaP module, followed by solvation in a truncated octahedral TIP3P water box with a 10 Å buffer, and neutralized using Na^+^ and Cl^−^ counterions. After generating topology and coordinate files, the system underwent two-stage energy minimization (2500 steps of steepest descent, followed by 2500 steps of conjugate gradient). Subsequent thermal equilibration involved gradual heating from 0 K to 298.15 K over 200 ps under NVT conditions, succeeded by 500 ps of NVT equilibration for solvent homogenization and 500 ps of NPT equilibration at 1 atm pressure. Temperature was controlled using the Langevin algorithm with a collision frequency of 2 ps^−1^, while pressure was maintained at 1 atm using the Berendsen barostat. The production phase comprised a 100 ns NPT simulation at 298.15 K and 1 atm with periodic boundary conditions applied throughout. A nonbonded cutoff of 10 Å was adopted, and the Particle Mesh Ewald (PME) method was utilized for long-range electrostatic interactions. The SHAKE algorithm was applied to constrain bonds involving hydrogen atoms with a time step of 2 fs. Trajectories were saved every 10 ps for subsequent analysis.

The binding free energies between the protein and ligand in all systems were calculated using the MM/GBSA method. In this study, the 90–100 ns MD trajectories were used for the calculations. The equation is as follows:

$${\Delta G}_{bind}={\Delta G}_{complex}-({\Delta G}_{receptor}+{\Delta G}_{ligand})\;\;\;\;={\Delta E}_{internal}+{\Delta E}_{VDW}+{\Delta E}_{elec}{+\Delta G}_{GB}+{\Delta G}_{SA}$$

where, ΔE_internal_ represents the internal energy, ΔE_VDW_ the van der Waals interactions, and ΔE_elec_ the electrostatic interactions.

The internal energy includes bond energy (E_bond_), angle energy (E_angle_), and torsional energy (E_torsion_). ΔG_GB_ and ΔG_SA_ are collectively referred to as the solvation free energy, where G_GB_ is the polar solvation free energy and G_SA_ is the nonpolar solvation free energy. For ΔG_GB_, the GB model developed by Nguyen et al. [23] was used in this study (*igb* = 2). The nonpolar solvation free energy (G_SA_) was calculated based on the product of the surface tension coefficient (γ) and the solvent-accessible surface area (SA), according to G_SA_ = 0.0072 × SASA. Entropic contributions were neglected in this study because of their high computational cost and low accuracy.

### 2.12. ACE Inhibition Kinetics

The inhibition mode of the peptides was elucidated by Lineweaver-Burk plot analysis, employing a previously reported method with minor modifications [24]. The assay was performed with FAPGG concentrations of 0.25, 0.5, 1, and 2 mM, and peptide concentrations of 0.25, 0.5, 1, and 2 mM. Double-reciprocal plots were constructed with the reciprocal of substrate concentration (1/[S]) as the x-axis and the reciprocal of reaction velocity (1/v) as the y-axis. The derived Lineweaver-Burk equations provided definitive classification of the inhibition type through their characteristic intersection patterns.

### 2.13. HUVECs Culture and Cell Viability Determination

Cell culture and cytotoxicity assessment were conducted according to the method described by Shufang Ye et al. with minor modifications [25]. Cryopreserved HUVECs were rapidly thawed in a 37 °C water bath, centrifuged to remove the freezing medium, and resuspended in fresh culture medium. The cells were seeded into 25 cm^2^ flasks and maintained at 37 °C with 5% CO_2_. Regular monitoring and subculturing were performed as needed.

The cytotoxicity profiles of four candidate peptides and Ang II were determined via CCK-8 assay in HUVECs. Following a 24 h adhesion period after seeding (1 × 10^4^ cells/well in 96-well plates), the test compounds were introduced at specified concentrations and incubated for a further 24 h. After the treatment period, cell viability was assessed by adding 10 μL of CCK-8 solution to each well, followed by a 2 h incubation. The absorbance at 450 nm was then measured using a microplate reader, and viability was calculated as a percentage relative to the untreated control groups. All experiments included six biological replicates per concentration to ensure statistical reliability.

### 2.14. Determination of NO and ET-1 Contents

HUVECs were seeded in 96-well plates at a density of 1 × 10^4^ cells per well and cultured for 24 h. The cells were then stimulated with 1 µM Ang II for 24 h, followed by treatment with the peptides at concentrations of 0, 50, 100, and 200 µM for another 24 h. NO levels in the collected supernatant were quantified by the Griess method, with measurements taken at 540 nm [26]. While ET-1 content was determined by an ELISA at 450 nm [27].

### 2.15. Statistical Analysis

All experiments were performed in triplicate. SPSS 24.0 software was used for statistical analysis. *p* < 0.05 was considered statistically significant. All charts were drawn by Origin 2018b.

## 3. Results

### 3.1. Ultrafiltration

Ultrafiltration is an effective method for fractionating enzymatic hydrolysates based on size, with distinct bioactivity differences observed between fractions. This finding is consistent with previous studies, which have demonstrated that antihypertensive peptides are generally present in fractions below 3 kDa [28]. The ACE inhibitory activity of the three ultrafiltration fractions (U1, U2, and U3) was determined. The results in Table 1 reveal a clear trend where both ACE inhibition and peptide content increased as molecular size decreased. The U3 fraction exhibited the strongest ACE inhibitory activity at 77.16%, with a peptide content of 68.82%. This value is significantly higher than those of the fractions with molecular weights greater than 3 kDa and those between 1 and 3 kDa (*p* < 0.05). These findings corroborate prior work by Li et al. [29], confirming that reduced molecular dimension enhances bioactive potential, likely due to improved steric accommodation within the catalytic pocket of ACE. The structure-activity relationship of ACE inhibitory food-derived peptides reveals that favorable inhibitory activity is associated with relatively short peptide chains, small molecular weights, and the appropriate spatial distribution of key amino acid residues that facilitate binding to the active site of ACE [30]. Hence, the U3 fraction was subsequently used for further purification.

### 3.2. Gel Filtration Chromatography

**Figure 1:**
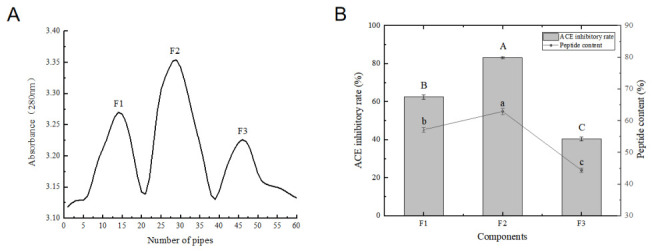
Gel filtration chromatography separation results with ACE inhibitory activity and peptide content. (**A**) Purification of the U3 fraction using a Sephadex G-15 column; (**B**) ACE inhibitory activity and peptide content of the obtained fractions. Note: Different letters (A–C; a–c) indicate significant differences (*p* < 0.05, Mean ± SD, *n* = 3).

As shown in Figure 1B, the F2 subfraction exhibited the strongest ACE inhibitory activity, achieving an ACE inhibition rate of 83.18% and a peptide content of 62.91%. Molecular mass analysis revealed that the peptides in all fractions were continuously distributed over a wide range of 200–1000 Da, with no obvious fraction-specific mass distribution. Notably, the smallest subfraction (F3) did not exhibit the highest inhibitory effect. This finding suggests that, although molecular size influences bioactivity, molecular weight alone does not determine ACE inhibitory potency. This likely occurs because the F3 fraction might contain more inactive short peptides or free amino acids. Additionally, the lower activity of some peptides with low molecular weights may be related to their amino acid sequences or spatial structural characteristics. These observations are consistent with the findings of Qiao et al. [31]. Collectively, the data indicate that ACE inhibitory activity is governed not only by molecular size but also by structural and compositional factors. Consequently, the F2 fraction was lyophilized for peptide sequencing.

### 3.3. Screening and Analysis of Potential ACE Inhibitory Peptides

**Figure 2:**
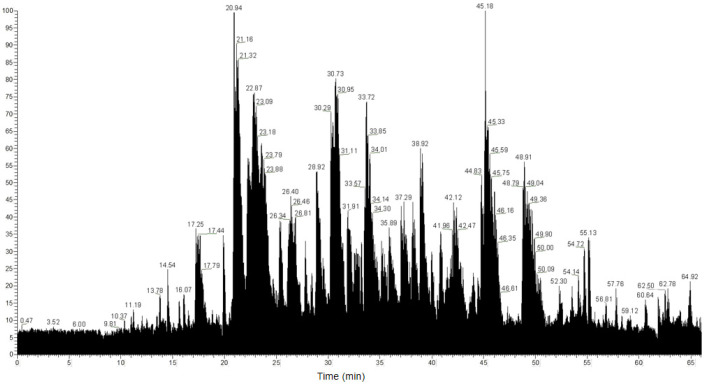
TIC chromatogram of the F2 fraction via LC−MS/MS. The x-axis represents retention time and the y-axis denotes ion intensity.

Liquid chromatography tandem mass spectrometry analysis of the F2 fraction identified 1980 unique peptides. The total ion chromatogram (TIC) shown in Figure 2 demonstrates favorable instrumental stability, low background noise, excellent chromatographic separation efficiency, and stable signal intensity throughout the analysis. The well distributed ion peaks further indicate the high complexity and good component separation of the peptide sample, providing reliable data support for subsequent peptide identification. The mass accuracy of the spectrometric analysis was verified by the mass error distribution (Figure S2), with most peptides showing mass errors within ±2 ppm and all within ±5 ppm. Peptide identification was performed with a false discovery rate of less than 1% to ensure high confidence. The LC–MS/MS identification data of the isolated active peptides has been publicly archived in the online repository to ensure experimental reproducibility (PXD076229).

These peptides predominantly ranged in length from 2 to 15 amino acids and exhibited diverse hydrophobicity profiles. The ACE inhibitory peptides were screened based on multiple criteria, including a bioactive score greater than 0.5, water solubility, stability, absence of allergenicity, and absence of toxicity, along with favorable predicted intestinal absorption efficiency, blood brain barrier permeability, and acute oral safety. According to the predictive results summarized in Table 2, four peptide sequences that meet the required conditions were identified, namely FHDYL, GFHLD, DLGHF, and NFGYDL. The MS2 spectra confirming the identification of these four peptides are provided in Figure S3. Their IC_50_ values were determined as 179.08, 207.61, 71.47, and 54.95 μM, respectively (Table 3). Although their ACE inhibitory activities were moderate compared with captopril, these peptides still exerted favorable and significant ACE inhibitory effects. For comparison, Mirdhayati et al. reported two ACE inhibitory peptides, EAPLNPKANR (IC_50_ = 44.6 μM) and IVG (IC_50_ = 97.3 μM), derived from Cangkuk, which demonstrated significant antihypertensive effects in spontaneously hypertensive rats after oral administration [32]. This finding implies a similar antihypertensive potential for these peptides.

Additionally, the ACE inhibitory activity of peptides is often influenced by their chain length. These four peptides were all composed of 5 to 6 amino acid residues. As noted by Liu et al., the limited spatial capacity of the ACE active site restricts the accommodation of large peptide molecules, and the number of amino acid residues directly affects the binding interaction between peptides and ACE and the inhibition efficacy [16]. This view is supported by Liao et al., who confirmed that peptides with high molecular weights generally exhibit difficulty in accessing the active site of the enzyme [33]. This also helps explain the relatively high ACE inhibition rates observed for these peptides. Moreover, systematic retrieval against the BIOPEP database, a widely used public repository for food-derived bioactive peptides, verified that the four identified peptide sequences have not been previously documented in the literature. For the purposes of this study, the term “novel ACE inhibitory peptides” is defined as peptides derived from bovine hemoglobin that meet two core criteria: no prior reports regarding their ACE inhibitory activity exist, and no corresponding entries are recorded in the BIOPEP database. Accordingly, four novel ACE inhibitory peptides with favorable bioactivity were successfully identified via liquid chromatography-tandem mass spectrometry coupled with in silico screening.

### 3.4. Molecular Docking

**Figure 3:**
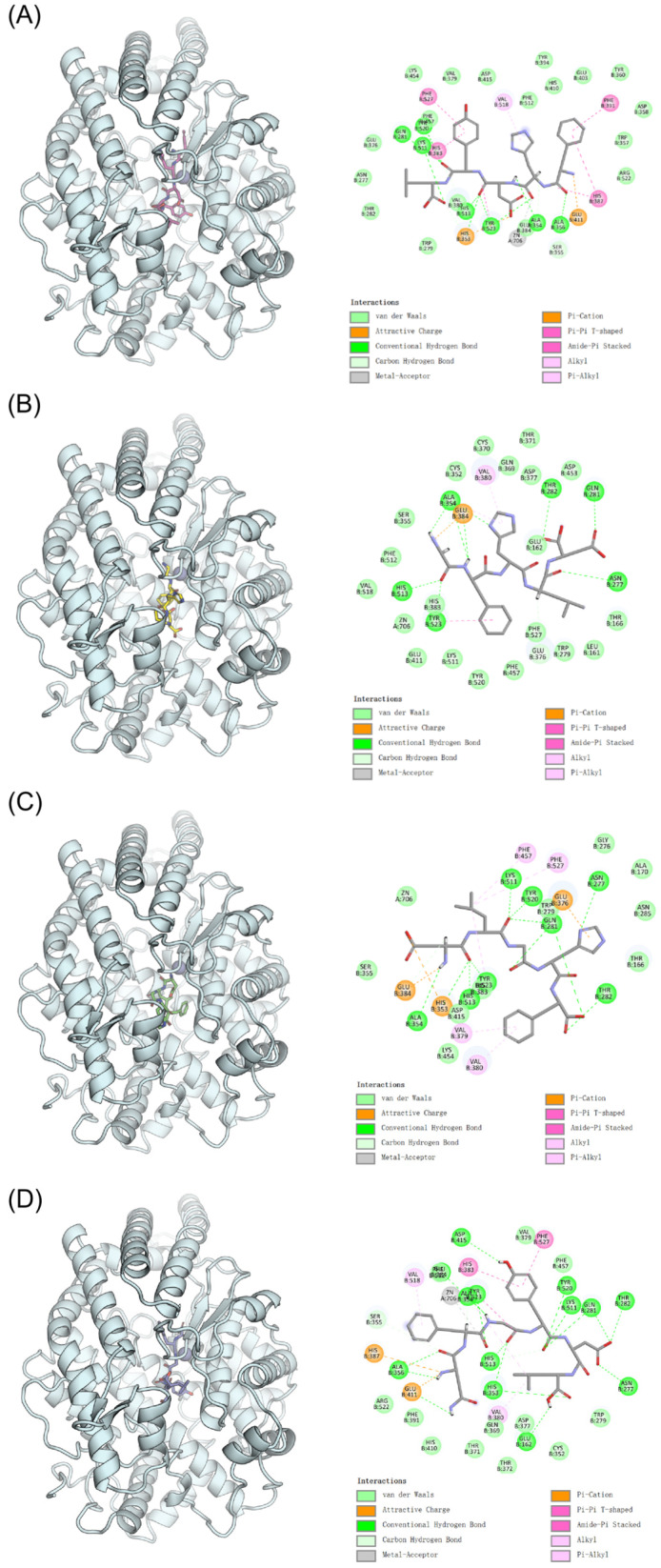
Molecular docking between four ACE inhibitory peptides and ACE active site. (**A**) FHDYL, (**B**) GFHLD, (**C**) DLGHF, (**D**) NFGYDL. Each group contains 3D binding conformations and 2D interaction profiles. The 3D view presents ACE (cyan cartoon), peptide (stick), and active-site Zn^2+^ (sphere). The 2D plots illustrate intermolecular interactions including hydrogen bonds, Pi-cation and van der Waals forces with surrounding amino acid residues.

Molecular docking with AutoDock Vina 1.2.3 revealed that the four inhibitory peptides bind to the active site of ACE, engaging the key substrate-binding pockets S1, S2, and S1′. The S1 pocket features key residues Ala354, Glu384, and Tyr523, while the S2 pocket contains Gln281, His353, Lys511, His513, and Tyr520. The S1′ pocket is characterized by the presence of Glu162. These structural features collectively govern the binding affinity and inhibitory potential of the identified peptides through specific molecular interactions [34]. The structural interactions between ACE and the identified peptides were visualized and analyzed using PyMOL 2.3.0 and Discovery Studio 2016 software. The MD model of ACE is shown in Figure S4. As depicted in Figure 3, the molecular docking results reveal distinct binding conformations for each peptide: (A) FHDYL, (B) GFHLD, (C) DLGHF, (D) NFGYDL. The protein structure is represented as a cyan cartoon model, while the peptides are displayed in stick representation. The left panel presents three-dimensional interaction views, clearly showing the spatial orientation of peptide binding, while the right panel provides complementary two-dimensional interaction diagrams detailing specific atomic-level contacts between the peptides and ACE active site residues.

Molecular docking analysis revealed that the peptide FHDYL forms seven hydrogen bonds with ACE residues Tyr520, Gln281, Lys511, Tyr523, His513, Ala354, and Ala356 (Figure 3A). Key interactions occur with Glu384 and Tyr523 in the S1 pocket, alongside Gln281, Lys511, His513, and Tyr520 in the S2 pocket. Furthermore, 18 van der Waals interactions were observed between FHDYL and ACE, further consolidating the binding stability of the complex [35]. For GFHLD (Figure 3B), five hydrogen bonds are formed within the ACE active site, engaging S1 residues (Ala354, Tyr523), S2 residue (Gln281), as well as Asn277 and Thr282. Similarly, peptide DLGHF forms eight hydrogen bonds with ACE residues Ala354, His513, His353, Gln281, Tyr520, Tyr523, Lys511, and Thr282 (Figure 3C). As a crucial non-covalent interaction, hydrogen bonding significantly contributes to the stability of the inhibitor-ACE complex [36]. Specifically, DLGHF effectively interacts with the S1 pocket (Ala354, Tyr523) and the S2 pocket (Gln281, His353, Lys511, His513, and Tyr520), which correlates with its favorable inhibitory capacity. Hydrogen bonds and van der Waals forces act as the primary stabilizing factors, potentially inhibiting ACE activity by inducing mild conformational changes within the active site. Additionally, electrostatic and hydrophobic interactions play essential roles in stabilizing the DLGHF-ACE complex, aligning with previous findings. However, the interaction network of this complex is primarily sustained by 20 van der Waals contacts, suggesting that hydrophobic interactions act as the dominant stabilizing force. Notably, the peptide NFGYDL exhibits strong inhibitory potency, which is closely associated with its extensive network of 13 hydrogen bonds with ACE (Figure 3D). Key interacting residues include Ala356, Asp415, Ala354, Tyr523, His513, His353, Glu162, Tyr520, Lys511, Gln281, Thr282, Asn277, and Glu384, which encompass almost all core residues across the three major binding pockets of ACE.

These results show that all four peptides bind to the catalytic Zn^2+^ center (Zn706) of ACE, which is supported by the residue interaction frequency data in Table 4. In native ACE, the Zn^2+^ ion adopts a stable distorted tetrahedral coordination geometry, maintained by three highly conserved residues: His383, His387, and Glu411. Upon docking, all four peptides preserve this intact coordination geometry without evident distortion. Through direct interactions with Zn706, they effectively occlude substrate access to the catalytic center, thereby establishing a robust structural basis for their antihypertensive effects. Table 4 summarizes the interaction frequencies of specific amino acid residues across the four peptide complexes. The catalytic Zn706 ion and its coordinating residues (His383, His387, and Glu411), together with Ser355, Gln281, Ala354, Glu384, Val379, Val380, His353, His513, Asn277, Phe457, Phe527, Tyr520, and Tyr523, consistently participate in binding across all four complexes. This pattern highlights their critical roles as core binding residues mediating ACE inhibitory activity. Remarkably, NFGYDL exhibits comprehensive interactions spanning all three canonical binding pockets of ACE: S1 (Tyr523, Glu384, Ala354), S2 (His513, Gln281, Tyr520, His353, Lys511), and S1′ (Glu162). This simultaneous occupancy of multiple binding pockets, combined with direct coordination at the Zn706 center, facilitates the formation of a highly stable complex between the enzyme and this peptide through synergistic noncovalent interactions. This multi-pocket engagement directly correlates with the superior ACE inhibitory activity of NFGYDL, as reflected by its significantly lower binding energy relative to the other three peptides (Table 4). Indeed, the concurrent occupancy of multiple substrate binding pockets and direct interaction with the catalytic Zn^2+^ ion appear to be critical determinants of enhanced binding affinity [37]. These findings are further corroborated by prior studies documenting analogous binding interactions between ACE and well-characterized inhibitory peptides such as PLITT, thereby reinforcing the mechanistic validity of the proposed binding mode [38].

In conclusion, the ACE-binding affinity of these identified peptides is governed by synergistic contributions from multiple molecular interactions. These include robust hydrogen bonding networks, extensive occupation of critical subsites (S1, S2, and S1′), optimized van der Waals contacts, and specific coordination with the catalytic Zn706 center. These structural insights effectively elucidate the structure-activity relationships of the target peptides and rationalise their favorable antihypertensive properties, providing valuable theoretical support for the rational design of novel ACE-targeted functional food ingredients and therapeutic candidates.

### 3.5. Molecular Dynamics Simulation of ACE-Peptide Complexes

**Figure 4:**
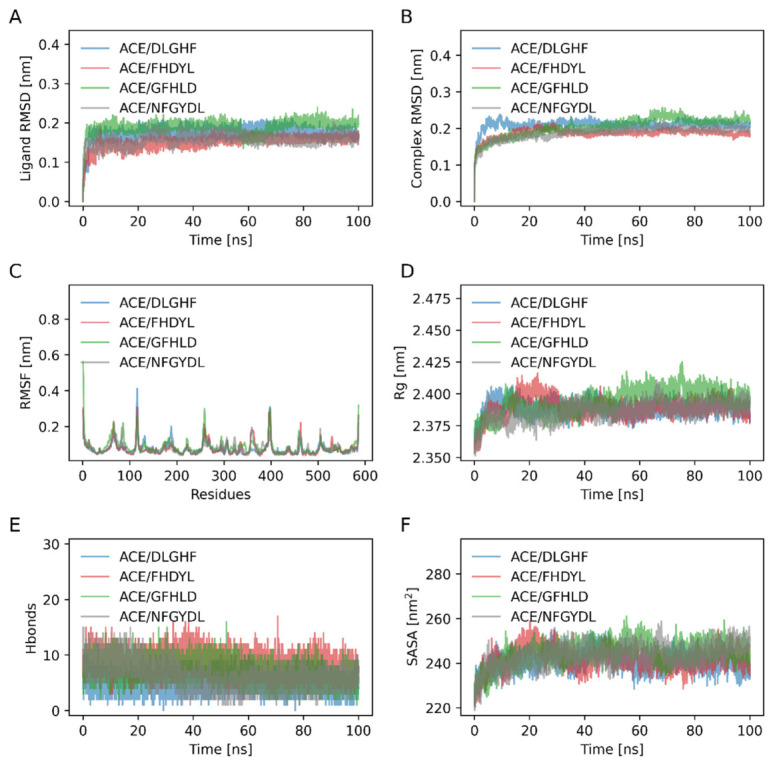
Time-dependent dynamic parameters of ACE-peptide complexes during 100 ns molecular dynamics simulation. (**A**) Ligand RMSD reflects peptide stability in the active pocket. (**B**) Complex RMSD indicates overall structural stability. (**C**) Protein RMSF shows residue flexibility of ACE. (**D**) Rg represents complex compactness. (**E**) Hydrogen bond number evaluates intermolecular interaction. (**F**) SASA reflects the hydration and stability of the complex.

The stability of the peptide-ACE complexes was assessed by RMSD, where smoother curves indicate higher conformational stability [39]. As shown in Figure 4A, the RMSD trajectories depict the conformational stability of each peptide within the ACE binding site. The four systems experienced a short period of adjustment initially and the equilibrium occurred, which shows that the peptides are well trapped in the binding pocket. As shown in Figure 4B, the average RMSD values post-equilibration for the ACE/DLGHF, ACE/FHDYL, ACE/GFHLD, and ACE/NFGYDL complexes were 0.2106 nm, 0.1962 nm, 0.1894 nm, and 0.2103 nm, respectively. Notably, the inter-complex differences in average RMSD values were minimal (approximately 0.02 nm), with no statistically significant divergence observed among the four systems. All systems reached stable convergence after roughly 10 ns, and the overall fluctuation ranges of RMSD values were narrow throughout the production phase, verifying the structural stability of all peptide-ACE complexes. The ACE/FHDYL complex showed the least total variation meaning that it is less perturbative to the protein structure when binding to the peptide. Conversely, the ACE/NFGYDL complex exhibited a stable range of fluctuations, indicating that the binding was not able to destroy the structure, but this may be a local conformational change as part of an established network of interactions.

RMSF analysis revealed consistent protein flexibility across all complexes, with elevated fluctuations localized primarily to the N-/C-terminal and specific loop regions. Slight differences in residue fluctuations near the binding site were observed, reflecting localized structural responses to peptide–ACE interactions. In particular, the ACE/NFGYDL complex showed relatively lower fluctuations within the binding region, suggesting that this peptide may effectively restrict local flexibility and thereby enhance the stability of the complex. The radius of gyration (Rg) serves as an indicator of structural compactness and molecular stability [3]. As shown in Figure 4D, the Rg profiles of all four complexes remained stable throughout the simulation, fluctuating within a narrow range centered around 2.39 nm. This consistency indicates that all four complexes maintained well-defined conformational compactness during the entire simulation period. Notably, the ACE/FHDYL and ACE/GFHLD complexes exhibited consistently low Rg values, confirming that both complexes adopted compact and stable structural configurations. In contrast, the ACE/NFGYDL complex displayed marginally elevated yet stable Rg values during the equilibrium phase. Rather than indicating structural looseness, this observation suggests that the complex achieves a dynamically balanced binding mode, likely facilitated by moderate conformational flexibility that enables optimal adaptation at the binding interface. To further investigate the hydrogen bonding characteristics within the binding sites, the number of key stabilizing hydrogen bonds between the compounds and the protein was analyzed [39]. Hydrogen bond analysis revealed that all complexes maintained a considerable number of hydrogen bonds throughout the simulation (Figure 4E). The ACE/FHDYL and ACE/GFHLD complexes exhibited relatively higher and more stable hydrogen bond counts, whereas ACE/NFGYDL showed moderate fluctuations while maintaining a satisfactory overall level of hydrogen bond occupancy. This pattern suggests that the binding of NFGYDL relies on a combination of multiple interaction types, and its dynamic hydrogen bonding profile may reflect a flexible binding mode rather than instability. The solvent-accessible surface area (SASA) describes the total biomolecular surface area accessible to solvent molecules and reflects conformational flexibility and compactness. SASA analysis revealed distinct structural dynamics among the peptide-ACE complexes, all systems underwent initial surface reorganization before reaching stable equilibria. The ACE/DLGHF and ACE/FHDYL complexes adopted more compact conformations, as evidenced by their reduced SASA values. In contrast, the ACE/NFGYDL complex exhibited moderately elevated yet stable SASA values, reflecting partial solvent exposure of its binding interface, a characteristic that may confer enhanced adaptability and dynamic equilibrium at the molecular level. Molecular dynamics simulations confirmed the robust structural stability of all peptide-ACE complexes, with distinct binding characteristics emerging among the different systems. The ACE/FHDYL and ACE/GFHLD complexes demonstrated superior structural compactness and extensive hydrogen bonding networks. In contrast, the ACE-NFGYDL complex exhibited a SASA value stabilizing at approximately 245 nm^2^, while displaying greater conformational flexibility yet maintaining remarkable stability. Notably, this conformational flexibility appears to facilitate enhanced binding adaptability and sustained molecular interactions, suggesting that NFGYDL employs a dynamic binding mechanism that combines structural plasticity with persistent intermolecular contacts. These findings highlight the significant potential of NFGYDL as a bioactive peptide ligand, where its unique balance of conformational adaptability and binding stability may contribute to improved functional performance. Belal et al. [40] systematically analyzed the molecular dynamics parameters, including root mean square deviation, root mean square fluctuation, and radius of gyration, for two representative synthetic inhibitors, captopril and lisinopril. Their results demonstrated that these synthetic inhibitors exhibited favorable binding stability. Comparative analysis indicated that the peptides identified in this study could also stably bind to ACE and effectively maintain the structural stability of the protein.

To reliably evaluate the binding affinities between the peptides and the target protein, binding free energies (ΔG_bind_) were calculated using the molecular mechanics generalized Born surface area method based on the molecular dynamics simulation trajectories. As summarized in Table 5, the ΔG_bind_ values for the ACE/DLGHF, ACE/FHDYL, ACE/GFHLD, and ACE/NFGYDL complexes were 22.43 ± 4.34, 45.10 ± 5.21, 34.72 ± 5.23, and 46.83 ± 11.07 kcal/mol, respectively. These favorable negative values indicate spontaneous and thermodynamically favorable binding of all four peptides to ACE. Notably, NFGYDL exhibited the lowest binding free energy, suggesting the strongest binding affinity among the evaluated peptides. Furthermore, energy decomposition analysis revealed that the binding interactions are predominantly driven by van der Waals forces and electrostatic interactions, with additional favorable contributions from nonpolar solvation free energy.

### 3.6. Inhibitory Kinetics Analysis

**Figure 5:**
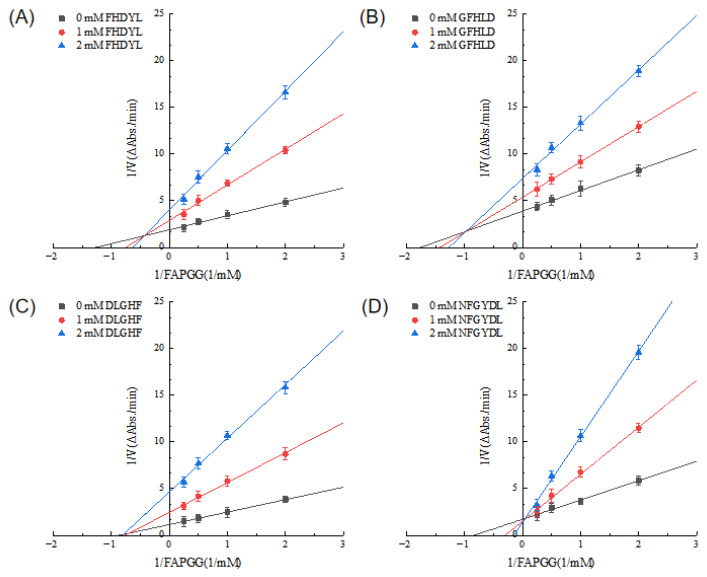
Lineweaver–Burk double reciprocal plots for identifying the inhibition types of four ACE inhibitory peptides. (**A**) FHDYL, (**B**) GFHLD, (**C**) DLGHF, (**D**) NFGYDL. The x-axis indicates 1/[FAPGG], and the y-axis denotes 1/V.

Kinetic characterization of the four bovine hemoglobin ACE inhibitory peptides revealed distinct ACE inhibition modes, as established by Lineweaver Burk plot analysis (Figure 5). NFGYDL exhibited a classical competitive inhibition pattern, in which the concentration dependent inhibition curves converged at the y intercept with successively increasing Km values. This kinetic profile indicates direct competition between the peptide and the substrate for the active site, with elevated Km values reflecting reduced substrate binding affinity. The consistent displacement of regression lines with increasing peptide concentration further supports the competitive inhibitory character of this hemoglobin derived peptide [41]. In contrast, both DLGHF and GFHLD exhibited characteristic noncompetitive inhibition kinetics, as evidenced by a concentration dependent reduction in Vmax while Km values remained constant. Similar behavior has been reported for non-competitive ACE-inhibitory peptides such as the highland barley peptide FPRPFL [42], longan seed peptides ETSGMKPTEL and ISSMGILVCL [43], oyster peptides NGDAGMV and EAGAGGL [44]. The kinetic behavior of FHDYL was consistent with mixed type inhibition, as demonstrated by distinct y intercept shifts at varying peptide concentrations accompanied by both decreased Vmax and increased Km values. This kinetic profile strongly suggests that FHDYL exerts its inhibitory effect through a dual binding mechanism: while capable of binding to the active site in competition with the substrate, it also interacts with an allosteric site, thereby inducing conformational changes in ACE that reduce its overall catalytic efficiency. Such simultaneous competitive and noncompetitive interactions represent a sophisticated mode of enzyme regulation that may provide more robust inhibition across different substrate concentrations under physiological conditions [45]. In recent years, several mixed-type ACE inhibitory peptides have been identified, such as the morel-derived peptide LIVPSLPGYAF, which similarly alters the enzyme’s active center conformation to block further substrate hydrolysis and achieve potent inhibition [46].

### 3.7. Cell Viability

**Figure 6:**
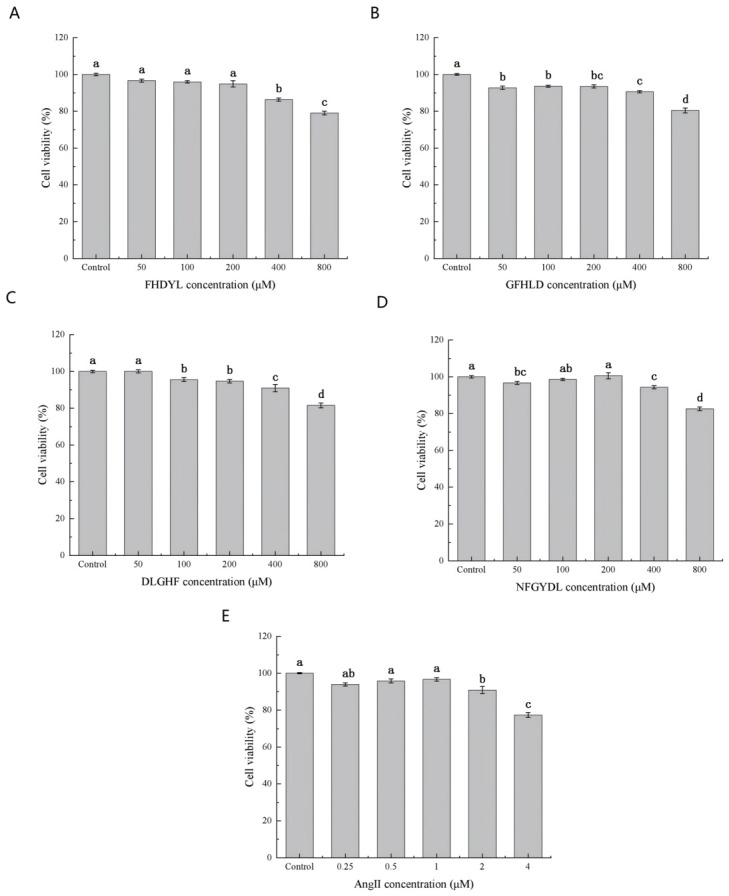
Effect of different concentrations of (**A**) FHDYL (**B**) GFHLD (**C**) DLGHF (**D**) NFGYDL and (**E**) Ang II proliferation of HUVEC. Different letters (a–d) indicate significant differences (*p* < 0.05, Mean ± SD, *n* = 6).

Vascular endothelial cells serve as a well-established cellular model for studying antihypertensive mechanisms [13]. The cytotoxicity of the four peptides and Ang II on HUVECs was evaluated via the CCK-8 assay, which revealed no significant cytotoxic effects, with cell viability consistently maintained above 90% across the entire tested concentration range (0–800 μM) for all peptide treatments (Figure 6A–D) [35]. These findings confirm the excellent biocompatibility of the investigated peptides within this physiologically relevant cell system, supporting their potential as safe bioactive compounds for further antihypertensive applications.

Considering the slight decrease in viability observed at higher concentrations (400–800 μM), subsequent experiments employed safe concentrations of 50, 100, and 200 μM. Ang II, a major inducer of hypertension, was used to establish a cellular injury model. Results in Figure 6E demonstrate that cell viability decreased significantly at Ang II concentrations exceeding 2 μM (77.36% at 4 μM), while remaining around 95% within the 0.25–2 μM range. Therefore, treatment with 1 μM Ang II for 24 h was selected as the experimental condition for inducing endothelial damage.

### 3.8. NO and ET-1 Contents

**Figure 7:**
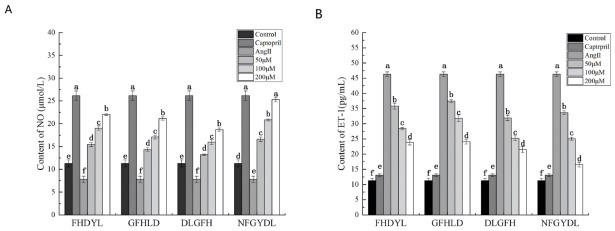
Effects of peptide treatments on NO production and ET-1 secretion in HUVECs. (**A**) NO production in HUVECs following treatment with the peptides FHDYL, GFHLD, DLGHF, and NFGYDL. (**B**) Secretion of ET-1 from HUVECs after exposure to the same peptides. Respectively, cells treated with captopril (Cap) served as the positive control. Different letters (a–f) indicate significant differences (*p* < 0.05, Mean ± SD, *n* = 6).

The regulation of vascular tone by endothelial cells is critically mediated through the balanced production of NO, a pivotal vasodilator essential for maintaining cardiovascular homeostasis, and ET-1, a potent vasoconstrictor. NO deficiency is strongly correlated with the development of hypertension and endothelial dysfunction [47], whereas excessive ET-1 production has been similarly implicated in the pathogenesis of vascular abnormalities and hypertensive conditions [26]. Nitrite quantification was performed using the Griess assay with a standard curve provided in Supplementary Figure S5. All four ACE inhibitory peptides derived from bovine hemoglobin demonstrated a dose dependent enhancement of nitric oxide production in HUVECs (Figure 7A). Notably, NFGYDL at a concentration of 200 μM exhibited comparable efficacy to the captopril positive control. This effect may be attributed to the known biological functions of ACE, which mediates the conversion of angiotensin I to angiotensin II and the degradation of bradykinin. Inhibition of ACE by these peptides may help preserve endogenous bradykinin levels, which in turn can interact with endothelial B2 receptors to promote nitric oxide production [2].

As evidenced in Figure 7B, all four peptides significantly suppressed endothelin 1 secretion in HUVECs, with NFGYDL and DLGHF at 200 μM exhibiting the most potent inhibitory effects. These findings are consistent with established evidence that ACE inhibitory peptides can simultaneously enhance nitric oxide bioavailability while attenuating ET-1 production [48]. The observed changes in ET-1 secretion may be associated with the ACE inhibitory activity of the peptides, which could indirectly influence the regulation of ET-1 expression [27]. Nevertheless, further investigations are warranted to fully elucidate the underlying molecular mechanisms. Collectively, these results demonstrate that the four ACE inhibitory peptides derived from bovine hemoglobin enhanced NO production and reduced ET-1 secretion in HUVECs, thereby improving endothelial function in vitro. These findings support the potential application of these peptides as bioactive ingredients in functional foods with demonstrable endothelial regulatory properties.

## 4. Conclusions

In summary, this study systematically isolated ACE inhibitory peptides from Yanbian cattle blood hydrolysates through a combination of ultrafiltration and gel filtration chromatography, with the F2 fraction demonstrating the highest bioactivity. Subsequent LC-MS/MS, coupled with comprehensive bioinformatic screening, led to the identification of four novel ACE inhibitory peptides: FHDYL, DLGHF, GFHLD, and NFGYDL. Among these, NFGYDL exhibited the most potent inhibitory activity, with an IC_50_ value of 54.95 μM. Enzyme kinetic analysis further classified FHDYL as a mixed-type inhibitor, NFGYDL as a competitive inhibitor, DLGHF and GFHLD as noncompetitive inhibitors. MD revealed that all four peptides exhibited high affinity binding at the ACE active site, while MD simulations confirmed the structural stability of the complexes formed between these peptides and ACE, as evidenced by low and converged values of key structural metrics, including RMSD, Rg, and RMSF, collectively indicating a compact and stable binding conformation. Furthermore, cellular assays conducted in HUVECs demonstrated that these peptides effectively modulated endothelial function by enhancing NO production and attenuating ET-1 secretion, thereby providing robust in vitro evidence for their ACE inhibitory and endothelial protective activities.

Looking ahead, future translational research will focus on further optimizing the identified peptides to enhance their pharmaceutical properties and broaden their application potential. Strategies including rational amino acid substitution, peptide cyclization, and terminal chemical modification will be systematically employed to strengthen ACE binding affinity and inhibitory potency. In parallel, the gastrointestinal stability and in vivo biosafety of these peptides will be rigorously evaluated. Moreover, advanced approaches such as microencapsulation and structural optimization will be explored to improve oral bioavailability and mitigate potential adverse effects. These efforts are expected to lay a solid scientific foundation for the development of these peptides as safe, stable, and efficacious bioactive ingredients for functional food applications.

## Figures and Tables

**Table 1:** ACE inhibitory activity and peptide content of ultrafiltered fractions with different molecular weight ranges (>3 kDa, 1–3 kDa and <1 kDa).

Molecular Weight	ACE Inhibitory Rate (%)	Peptide Content (%)
>3 kDa	41.99 ± 0.51	49.37 ± 0.3
1–3 kDa	57.23 ± 0.58	66.53 ± 0.26
<1 kDa	77.16 ± 0.76	68.82 ± 0.09

Data are presented as mean ± standard deviation (*n* = 3), showing the in vitro ACE inhibition rate and total peptide content of each fraction.

**Table 2:** Predicted physicochemical characteristics and bioactivities of four identified ACE inhibitory peptides.

Predicted Parameters	FHDYL	GFHLD	DLGHF	NFGYDL
Molecular Weight	693.3122	587.2703	587.2703	727.3177
−10 lgP	42.1	61.95	65.05	95.68
PeptideRanker	0.810761	0.651367	0.790307	0.821164
gravy	−0.28	−0.1	−0.1	−0.35
instability_index	8	8	−12.74	8.6
pLM4Alg	Non-Active	Non-Active	Non-Active	Non-Active
Toxin	Non-Active	Non-Active	Non-Active	Non-Active
Docking energy (kcal/mol)	−10.54	−9.206	−9.411	−11.179
HIA	0.068	0.055	0.025	0.094
BBB	0.043	0.263	0.275	0.035
ROA	0.205	0.085	0.085	0.112

**Table 3:** IC_50_ values of four kinds of ACE inhibitory peptides and positive-control captopril.

Sample	IC_50_ (μM)
FHDYL	179.08
GFHLD	207.61
DLGHF	71.47
NFGYDL	54.95
Captopril	0.025

**Table 4:** Interacting amino acid residues between the four ACE inhibitory peptides and ACE. “+” indicates detectable intermolecular interactions, while blanks represent no obvious binding.

	FHDYL	GFHLD	DLGHF	NFGYDL
Zn706	+	+	+	+
Ser355	+	+	+	+
Gln281	+	+	+	+
Ala354	+	+	+	+
Ala356	+	+		+
Glu384	+	+	+	+
Glu376	+	+	+	
Glu411	+	+		+
Val379	+	+	+	+
Val380	+	+	+	+
Val518	+	+		+
His353	+	+	+	+
His513	+	+	+	+
His383	+	+	+	+
His410	+	+		+
His387	+	+		+
Asn277	+	+	+	+
Phe457	+	+	+	+
Phe527	+	+	+	+
Phe391	+	+		+
Phe512	+	+		+
Thr282	+	+		+
Trp279		+	+	+
Tyr520	+	+	+	+
Tyr523	+	+	+	+
Lys511	+	+		+
Asp415	+	+		+

**Table 5:** Binding free energies and energy components predicted by MM/GBSA (kcal/mol).

System Name	ACE/FHDYL	ACE/GFHLD	ACE/DLGHF	ACE/NFGYDL
**Δ*E*_vdw_**	−65.00 ± 5.16	−51.02 ± 3.45	−66.40 ± 1.41	−51.85 ± 4.51
**Δ*E*_elec_**	−73.88 ± 18.71	−74.32 ± 10.95	−52.21 ± 10.92	−60.90 ± 18.19
**ΔG_GB_**	103.54 ± 17.84	98.59 ± 12.00	105.14 ± 8.05	75.74 ± 16.29
**ΔG_SA_**	−9.75 ± 0.10	−7.97 ± 0.17	−8.95 ± 0.06	−9.82 ± 0.26
**ΔG_bind_**	−45.10 ± 5.21	−34.72 ± 5.23	−22.43 ± 4.34	−46.83 ± 11.07

ΔEvdW: van der Waals energy. ΔEelec: electrostatic energy. ΔGGB: electrostatic contribution to solvation. ΔGSA: non-polar contribution to solvation. ΔGbind: binding free energy.

## Data Availability

The original contributions presented in this study are included in the article/Supplementary Materials. Further inquiries can be directed to the corresponding author.

## Supplementary Materials

The following supporting information can be downloaded at: https://www.mdpi.com/article/10.3390/foods15081414/s1, Figure S1. Standard curve for the determination of peptide content by OPA method. The x-axis represents standard peptide concentration, and the y-axis denotes absorbance at 570 nm; Figure S2. Mass error distribution of identified peptides from the F2 fraction via LC−MS/MS. The x-axis represents mass error (ppm), and the y-axis denotes the peptide number; Figure S3. MS/MS spectra of peptides FHDYL, GFHLD, DLGHF and NFGYDL. The x-axis represents the mass-to-charge ratio (*m*/*z*), and the y-axis denotes relative ion intensity (%); Figure S4. Molecular docking model of the identified peptide with ACE; Figure S5. Standard curve for nitrite quantification using the Griess assay. The x-axis represents sodium nitrite concentration, and the y-axis shows absorbance at 540 nm.

## Abbreviations

The following abbreviations are used in this manuscript:

ACE	Angiotensin-converting enzyme
HUVEC	Human umbilical vein endothelial cells
LC-MS/MS	Liquid chromatography-tandem mass spectrometry
MD	Molecular docking
NO	Nitric oxide
ET-1	Endothelin-1
RMSD	Root mean square deviation
RMSF	Root mean square fluctuation
Rg	Radius of gyration
ELISA	Enzyme-linked immunosorbent assay
SHR	Spontaneously hypertensive rat
SASA	Solvent-accessible surface area
DDA	Data-dependent acquisition
OPA	O-phthaldialdehyde
HIA	Human intestinal absorption
ESI	Electrospray ionization
HCD	Higher-energy collisional dissociation
IAM	Iodoacetamide
