# Supplementary material for: ACE-Inhibitory Peptides from Yanbian Cattle Hemoglobin: Screening, Kinetics, and Molecular Dynamics Simulation
Source: Foods. 2026 Apr 17;15(8):1414. doi: 10.3390/foods15081414 (PMC13115288; doi:10.3390/foods15081414)
Supplement: Supplementary file 1 [file foods-15-01414-s001.zip › foods-4209816-supplementary.pdf]

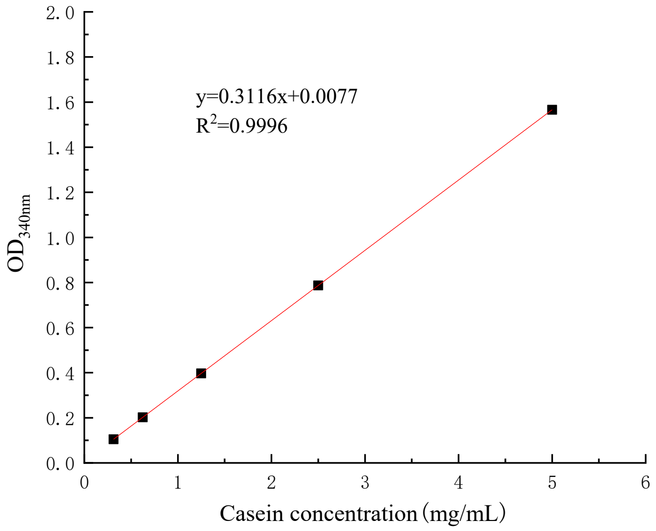

Figure S1. Standard curve for the determination of peptide content by OPA method. The x-axis represents standard peptide concentration, and the y-axis denotes absorbance at 570 nm.

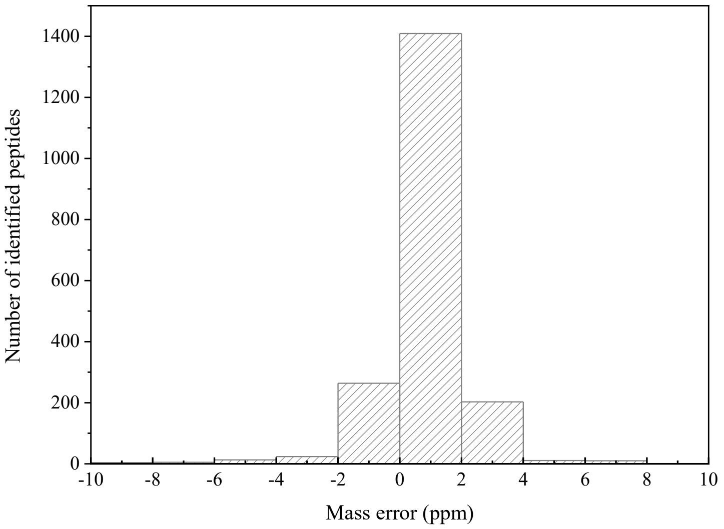

Figure S2. Mass error distribution of identified peptides from the F2 fraction via LC–MS/MS. The x-axis represents mass error (ppm), and the y-axis denotes the peptide number.

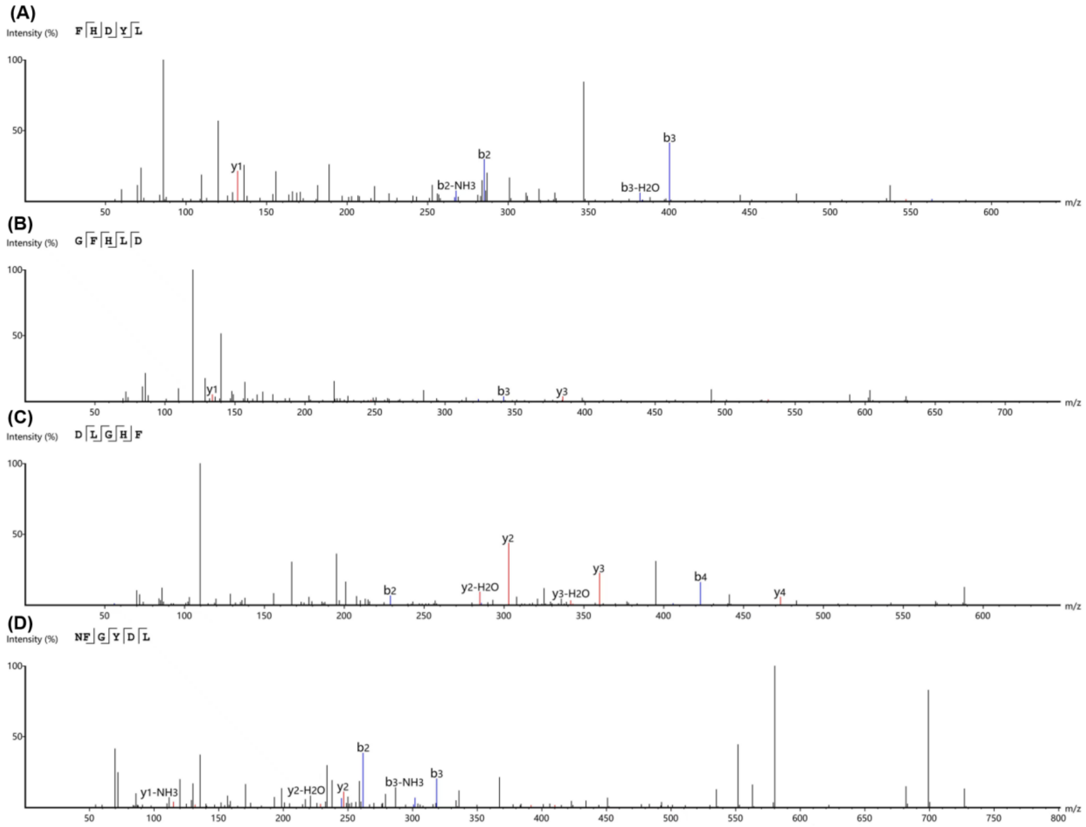

Figure S3. MS/MS spectra of peptides FHDYL, GFHLD, DLGHF and NFGYDL. The x-axis represents the mass-to-charge ratio (m/z), and the y-axis denotes relative ion intensity (%).

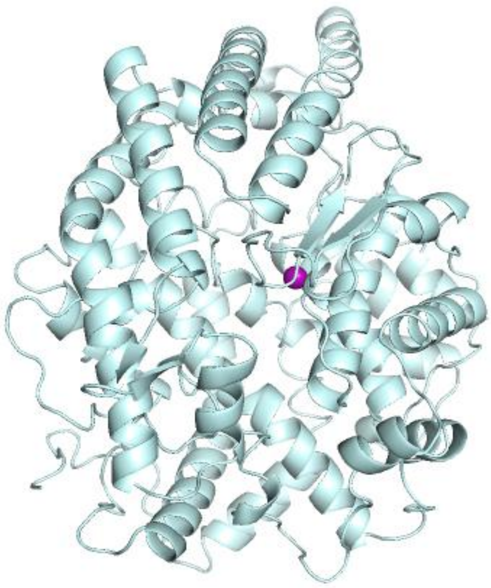

Figure S4. Molecular docking model of the identified peptide with ACE.

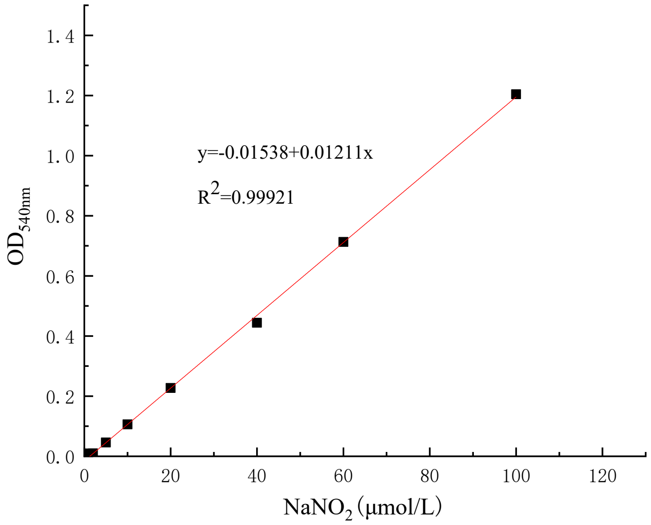

Figure S5. Standard curve for nitrite quantification using the Griess assay. The x-axis represents sodium nitrite concentration, and the y-axis shows absorbance at 540 nm.
